# Hallmarks of human “immunosenescence”: adaptation or dysregulation?

**DOI:** 10.1186/1742-4933-9-15

**Published:** 2012-07-25

**Authors:** Graham Pawelec

**Affiliations:** 1 Center for Medical Research, University of Tübingen Medical School, Waldhörnlestr. 22, Tübingen, D-72072, Germany

## Abstract

Is immunosenescence an intrinsic ageing process leading to dysregulation of immunity or an adaptive response of the individual to pathogen exposure? Age-associated differences in bone marrow immune cell output and thymic involution suggest the former. Accepted hallmarks of immunosenescence (decreased numbers and percentages of peripheral naïve T cells, especially CD8 + cells, and accumulations of memory T cells, especially late-stage differentiated CD8+ cells) suggest the latter, viewed as the result of depletion of the reservoir of naïve cells over time by contact with pathogens and their conversion to memory cells, the basis of adaptive immunity. Thymic involution beginning early in life limits the generation of naive cells such that the adult is believed to rely to a great extent on the naïve cell pool produced mostly before puberty. Thus, these hallmarks of immunosenescence would be markedly affected by the history of the individual´s exposure to pathogens. It would be predicted that in modern industrialized populations, the cumulative effects of antigenic “stressors” would be lower than in less hygienic societies, whereas intrinsic processes might be more similar in different populations. Identifying such stressors and taking steps to nullify their impact could therefore result in delayed immunosenescence and contribute significantly to improving public health. Here, I discuss some of the available data bearing on this prediction.

## How can we define and measure “immunosenescence”?

To answer any questions on immunity and ageing, we clearly need to have a means to measure immunosenescence. At the moment, we only have rather unrefined biomarkers which we believe may reflect detrimental effects of age. Traditionally, cross-sectional studies in industrialized countries have almost unanimously documented lower numbers and/or proportions of peripheral blood naïve CD8+ T cells in the elderly, whereas age-effects on CD4+ T cells have been harder to pin down. Reciprocally, memory CD8 + T cells are more numerous in the elderly, again with lesser and more discordant effects reported for CD4 + T cells [1-3]. It has been the assumption that the greater susceptibility of the elderly than the young to novel infections reflects this lesser availability of naïve cells poised to recognize new antigens. To the best of my knowledge, however, this assumption has never been experimentally tested in humans.

Based on a series of prospective cohort studies of the very elderly in Sweden, the concept of an “immune risk profile” (IRP), however, has gained traction over the past decade. The IRP was found to be present in around 15% of 85-year-olds in the OCTO/NONA studies at baseline [4,5]. Follow-up of 2-, 4- and 6-year mortality revealed significantly higher all-cause mortality in the IRP group than in the majority of very elderly. The IRP was characterized by a relative deficit in the numbers and proportions of B cells, and an accumulation of CD8+ memory T cells tipping the CD4:CD8 ratio to less than unity (in contrast to the majority of elderly where the CD4:CD8 ratio *increases* rather than decreasing). This accumulation of late-stage differentiated cells (double-negative for the costimulatory receptors CD27 and CD28) was responsible for the poor T cell proliferative responses to mitogens which was characteristic of the cluster of parameters constituting the IRP, as the majority of these cells were later found also to express KLRG-1 and CD57 (ie., double-positive for negative regulatory coreceptors). This phenotype, or more commonly simply CD8 + CD28-negative, is often taken to mark senescent cells; I would argue strongly against this, although some of them might indeed be senescent. Caution would indicate designating them only as “late-stage differentiated”. It should be noted here that the numbers and proportions of CD8+ naïve cells were NOT part of the IRP, being greatly reduced in both groups. Additionally, neither naive or memory CD4+ T cells featured in these studies as informative for all-cause mortality [4,5].

The final parameter of the original IRP was, remarkably, Cytomegalovirus seropositivity. Although this persistent herpesvirus infected 85 % of the whole elderly population, every individual in the IRP group was a carrier. Thus, CMV-seropositivity was a part of the IRP; however, most CMV-infected people were nonetheless not in the IRP, already clearly indicating that CMV is a contributing but not controlling factor [6-9]. Studies of 2-, 4- and 6-year survival in these cohorts also revealed a second set of parameters, *independent* of the IRP, but at least additive with the IRP, which associated with mortality. These factors were higher levels of the pro-inflammatory cytokine IL 6, associated with frailty and mortality in countless studies, together with measures of cognitive impairment. Because some of the literature refers to the “IRP” as also including IL 6, I stress here that the only studies documenting the original IRP did NOT include IL 6. Thus survival of people with lower IL 6 and not in the IRP was better than those with the IRP alone, or high IL 6 alone, and much better than people in the IRP who also had high IL 6 [4,5]. The literature also includes many papers referring to the presence or absence of the IRP in different populations and situations. Again, I emphasize that the only studies showing the IRP to be informative for mortality are the Swedish OCTO/NONA studies. Whether this is also true of other populations has not been tested. Moreover, we do not know at which calendar age the IRP may become predictive of mortality; very preliminary data suggests possibly 60 years of age [10]. At much older ages, the IRP is again not predictive, in that no subjects surviving long enough are found to be in the IRP category, presumably due to selection of those who were [11]. Consistent with this, we have recently completed a small-scale study of subjects in the Leiden 85-Plus study, which as the name suggests also started from a baseline of 85 [12]. Here, we analyzed subjects at the age of 89 and found that only one was in the IRP. Thus, in the highly selected very elderly population, one would not necessarily expect to find anyone still in the risk group. Fascinatingly, in the Leiden 85-Plus study, we found that a *lower* proportion of naïve CD8+ T cells and a *higher* proportion of some CD8+ memory cell phenotypes was associated with longer survival on 8-year follow-up [12]. Clearly, greater naïve cell capacity under these conditions was not associated with favourable outcome. We interpret these data to suggest that in a very elderly population presumably not exposed to many new pathogens, it is more important to maintain good memory responses rather than use reserves to meet new challenges that may never come. The finding that longer survival was also associated with in unopposed pro-inflammatory responsiveness to CMV antigens in vitro [12] underlines the nature of the memory responses that we propose are essential to maintain in old age. Thus, these considerations emphasize that immune parameters associated with survival maybe very different in different populations at different ages. This makes it unlikely that general “one size fits all” biomarkers of immune ageing (or any other parameter for that matter) will be identified. A consensus view would be that low naïve cells, high memory cells and high pro-inflammatory states would mitigate against survival. However, it is clear that in certain cases, such as the example of the Leiden 85-Plus described above, the exact opposite seems to be true. Other exceptional populations, such as centenarians, may also need to be considered separately [13-15].

## Genetic variables determining longevity?

Of the many variables potentially influencing longevity and biomarkers of ageing, the genetic background certainly represents a likely suspect. Huge amounts of resources have been and are being expended in searching for genetic and epigenetic influences on human longevity. Relatively little exploration of immune parameters is so far available in this respect, but data emerging from a study of familial longevity may shed some light on this matter. The Leiden Longevity Study (LLS), in which the 40-80-year-old subjects, representing 0.5 % of the general population, enjoy a 30 % decreased standardized mortality rate, is a very valuable resource in this respect. It seems that the potentially long-lived offspring in the LLS do not show the characteristically decreased proportions of CD8+ naïve cells, nor the accumulations of CD8+ late memory cells [16]. A remarkable finding in these individuals was that even if they were infected with CMV, their naïve cells were not “used up”, in contrast to the normal population. It has become very clear since the first reports of the impact of CMV on immune parameters [17,18] that it is the fact of infection with this persistent virus that is to a large degree responsible for the shifts in the proportions of CD8+ naïve and memory cells. Thus, in cross-sectional studies of people of different ages, those who are CMV-negative do not show loss of naïve cells at all, or to nothing like to the same degree [16,19]. The fact that CMV actually *causes* these shifts is made highly likely by findings from allograft transplantation where a CMV + organ causes infection of a CMV-negative recipient, with rapid loss of naïve cells and accumulation of memory cells [20]. Hence, in people genetically predisposed to longer life, the lack of the requirement for the huge amount of immune resources otherwise taken up with CMV immunosurveillance throughout life may provide an advantage in itself. This in turn would suggest that people in the general population who remain CMV-negative might also have a survival advantage. There is indeed some evidence for this from several studies, including the large NHANES survey of 14,000 adults over 10 years [21,22].

## Does immunosenescence occur earlier in life in developing countries?

Most studies attempting to assess immunosenescence or biomarkers of immune ageing have focused on a single population, mostly “WEIRD” (ie Western, educated, industrialized, rich, democratic). These are not necessarily representative of the majority off the world´s population. There will not only be differences in genetics, the effects of which may be wide-ranging, as described above for the LLS, but also in societal and psychological factors, and perhaps most importantly in nutritional status and access to health care. One very striking difference is also that fact that in the “wild-type” situation, all humans are infected with CMV from the age of ca. 2 months on, when they no longer receive only anti-CMV antibody in the mother´s milk, but also the infectious virus that has reactivated in the meantime. CMV-negativity is an artifact of civilization, hygiene and decreased breast feeding. Hence, in our pilot study of young and old men in rural Pakistan, all the young were already CMV-positive [23]. As ”old” is viewed as >50 years in this society, we sought to establish whether age-associated differences in immune phenotypes that we and others had established in older European and US populations were similar in Pakistanis, and whether they manifested earlier in the latter. We concluded that there were two major differences between the Pakistani population and the historical controls of WEIRD subjects. One was that we did indeed see age-associated differences in CD8+ T cells earlier in the Pakistanis, and the other was that we saw for the first time in a healthy population that not only the CD8+ subset but also the CD4 + T cells were affected. This we had otherwise only seen in pathological European populations, eg. those with Alzheimer´s [24]. We interpret this to mean that the level of “antigenic stress” in the Pakistani population, old a 50, could indeed be leading to “premature immunosenescence”.

## Conclusions

As far as we know from animal studies, bone-marrow output of immune cells is affected by age, but varies greatly dependent on genetic background. The general finding, though, is that output of myeloid cells increases and B and T cell progenitors decreases. Whether this is an intrinsic process reflected in the decreased DNA repair capacity of the compensatorily increased cycling stem cells is not understood. Why the thymus involutes as part of a developmental not senescing process is also not understood. However, against this backdrop of altered immune cell production, it seems safe to argue that processes directly responsible for immunosenescence are to a great extent determined by that individual´s history of pathogen exposure, and in humans, particularly to infection with CMV. There is therefore also an argument for medical intervention to offset the impact of chronic antigenic stress on the immune system, which in developing countries would certainly include CMV, which infects essentially the entire population. In industrialized countries, where CMV infection rates are probably still decreasing, different, so-far unidentified pathogens in CMV-negative individuals may need to be targeted [25].

## Competing Interests

The author declares that he has no competing interest.
